# Dissecting causal relationships between gut microbiota, blood metabolites, and glioblastoma multiforme: a two-sample Mendelian randomization study

**DOI:** 10.3389/fmicb.2024.1403316

**Published:** 2024-07-03

**Authors:** Xuan Chen, Lihui Han, Wenzhe Xu

**Affiliations:** ^1^Department of Radiation Oncology, Qilu Hospital of Shandong University, Jinan, China; ^2^Department of Neurosurgery, Qilu Hospital of Shandong University and Institute of Brain and Brain-Inspired Science, Shandong University, Jinan, China; ^3^Shandong Key Laboratory of Brain Function Remodeling, Jinan, China

**Keywords:** glioblastoma, gut-brain axis, gut microbiota, Mendelian randomization, metabolites

## Abstract

**Background:**

Given the increasing interest in the role of gut microbiota in glioblastoma multiforme (GBM), our objective was to examine the potential causal relationship between gut microbiota and GBM, as well as the mediating effects of specific metabolites.

**Methods:**

A bidirectional two-sample Mendelian randomization (MR) analysis was conducted to investigate the associations between 196 microbial taxa and GBM. A two-step MR technique was used to identify significant mediators in this relationship. Subsequently, a mediation analysis was performed to explore and quantify the mediating effects of specific metabolites on the causal relationship between gut microbiota and GBM.

**Results:**

Five taxa showed significant associations with GBM. Among them, *family Victivallaceae* [odds ratio (OR): 1.95; 95% confidence interval (CI): 1.21, 3.13; *p* = 0.005] and *genus Lactococcus* (OR: 1.81; 95% CI: 1.04, 3.15; *p* = 0.036) were positively correlated with the risk of GBM, while *phylum Cyanobacteria* had a protective effect against GBM (OR: 0.45; 95% CI: 0.22, 0.89; *p* = 0.021). The mediation analysis revealed that the connections among *family Victivallaceae, genus Lactococcus, phylum Cyanobacteria* and GBM were mediated by Methyl-4-hydroxybenzoate sulfate, phosphoethanolamine and dehydroepiandrosterone sulfate. Each of these accounted for 7.27, 7.98, and 8.65%, respectively.

**Conclusion:**

Our study provides evidence supporting a potential causal association between certain gut microbiota taxa and GBM. The study highlights the central role of gut microbiota in GBM pathogenesis and their interactions with vital serum metabolites. This paves the way for potential novel therapeutic interventions in GBM management.

## 1 Introduction

The gastrointestinal tract represents the largest mucosal surface area of the human body and is consistently subjected to a multitude of antigens and microorganisms in the daily diet (Tilg et al., 2022). A growing body of evidence suggests that the gastrointestinal tract and associated microbiota engage in a complex and integrated dialogic mechanism, as evidenced by numerous recent studies. The gut microbiome performs a variety of noteworthy biological functions, including regulating nutrient harvest from the diet, maintaining intestinal barrier integrity, metabolizing cholesterol, converting bile acids, producing antimicrobial peptides, metabolizing drugs, and influencing immunity and autoimmunity (Loh et al., 2024). In addition to the digestive tract, the intestinal flora is known to communicate with various organs, such as the liver, lungs, and central nervous system (CNS), through a variety of pathways, including the gut-liver axis, the gut-lung axis, and the gut-brain axis (Budden et al., 2017; Agirman et al., 2021; Pabst et al., 2023). These interactions are becoming increasingly recognized as crucial for maintaining overall organ health.

Glioblastoma multiforme (GBM) is the most common and deadly type of brain tumor. It is characterized by fast cell growth, active vascularization, significant heterogeneity, and extensively infiltration (Kumari and Kumar, 2023). Despite an aggressive standard treatment, which includes maximal safe surgical removal, radiation therapy, and temozolomide chemotherapy, GBM patients have a poor median survival time of only 14.6 months (Stupp et al., 2005). Therefore, there is an urgent need to develop additional therapeutic strategies and investigate the mechanisms that contribute to the development and progression of GBM.

Recent studies have suggested a correlation between gut microbiota and GBM pathogenesis (Mehrian-Shai et al., 2019; D'Alessandro et al., 2021; Zhang et al., 2022). The microbial composition in healthy individuals differs significantly from that of glioma patients (Jiang et al., 2022). Additionally, antibiotic treatment has been shown to alter the composition of intestinal microbiota and promote GBM growth (D'Alessandro et al., 2020). The gut-brain axis, a bidirectional pathway, establishes a connection between gut microbiota and the CNS (Mayer et al., 2022). The gut microbiota produces and consumes various molecules, such as peptides, neurotransmitters, and neuroactive substances, which travel through the bloodstream to reach the brain (D'Alessandro et al., 2021; Liu et al., 2022). Microbiota-derived metabolites act as signaling molecules that regulate the maturation of immune cells in CNS and the entire body, thereby modulating the GBM microenvironment (Dehhaghi et al., 2020).

Clinical and animal studies have shown that gut microbiota may affect GBM by modulating the blood levels of some bioactive metabolites, such as glutamate and short-chain fatty acid (SCFA) (Lyu et al., 2022). Therefore, we aimed to investigate the causal associations between gut microbiota, metabolites, and GBM. Our goal was to identify potential metabolites that could be used for early diagnosis and as clinical treatment targets. Mendelian randomization (MR) is a widely accepted method for controlling potential confounding factors and avoiding reverse causation bias when inferring causality between exposure and clinical outcomes (Emdin et al., 2017). This is achieved by using genetic variants as instrumental variables (IVs). MR has been widely applied to evaluate the potential causal association between gut microbiota and disease-risking genes (Ni et al., 2021; Xu et al., 2021; Ma et al., 2023a). This study utilized bidirectional MR analysis and mediation analysis with summary statistics from the latest genome-wide association studies (GWAS) of the gut microbiota, blood metabolites, and GBM to investigate their associations.

## 2 Materials and methods

### 2.1 Study design

**Figure 1:**
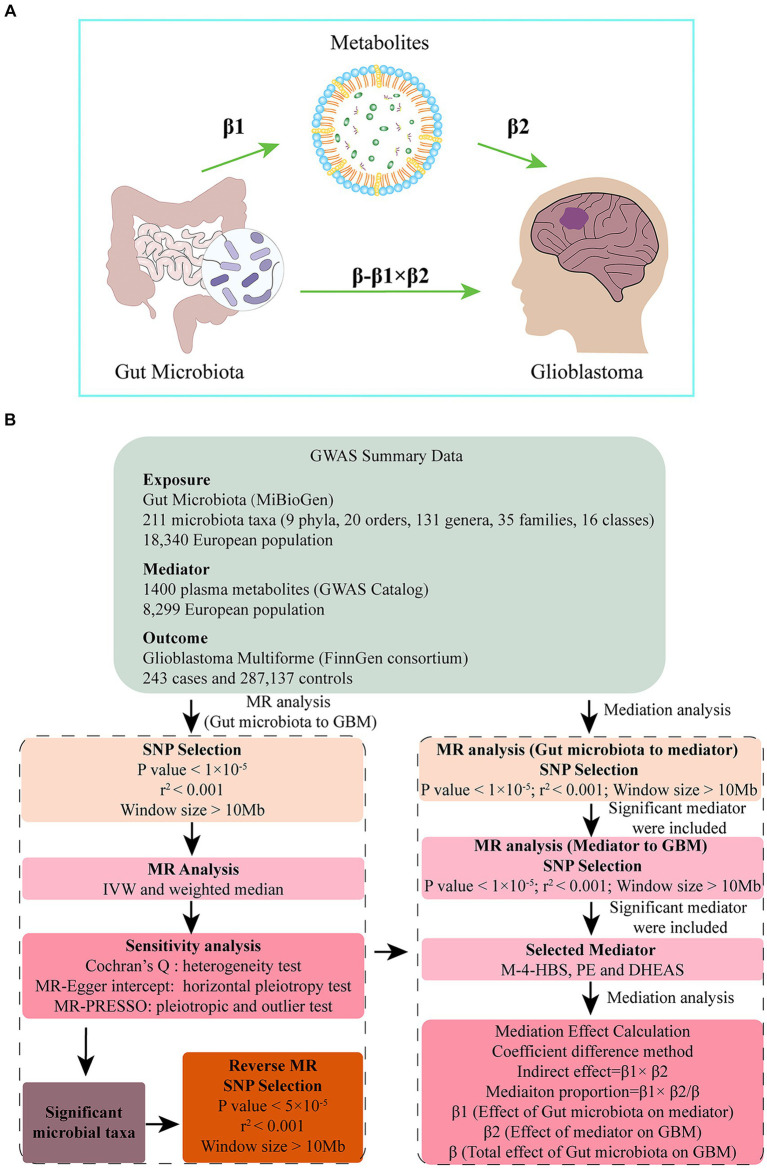
**(A)** Diagrams illustrating associations examined in this study. The total effect (β) was decomposed into: (i) indirect effect (β1 × β2) (where β1 is the effect of gut microbiota on metabolite, and β2 is the effect of metabolite on GBM) and (ii) direct effect (β-β1 × β2). Mediated proportion was the indirect effect divided by the total effect. **(B)** Flow chart outlining the methodology used to investigate the relation between gut microbiota and GBM. Mediation analysis evaluated the potential influence of M-4-HBS, PE and DHEAS on the microbiota-GBM association.

This study is reported following the Strengthening the Reporting of Observational Studies in Epidemiology Using Mendelian Randomization guidelines (STROBE-MR, S1 Checklist) (Skrivankova et al., 2021). A two-sample MR was conducted to investigate the causal association between gut microbiota and GBM using genetic variants, specifically single nucleotide polymorphisms (SNPs), as IVs. To ensure the directionality and robustness of the results, reverse MR and sensitivity analysis were performed. To account for the intricate interaction between metabolism and gut microbiota, 1,400 metabolites were selected as potential mediators. We applied a two-step MR approach to identify significant mediators. Then we calculated the proportion of the effect of gut microbiota on GBM that is mediated through specific metabolites. This allowed us to estimate the direct effect of gut microbiota on GBM, adjusting for the significant mediators. Figure 1B presents a flow chart illustrating our study design and analytical steps.

### 2.2 Data sources

Summary statistics for human gut microbiota (*n* = 18,340) were obtained from the MiBioGen study, which is the largest multi-ethnic GWAS meta-analysis of the gut microbiome to date (Kurilshikov et al., 2021). The GWAS Catalog was used to obtain summary statistics for plasma metabolites (*n* = 8,299) (Accession number: GCST90199621-90201020). The levels of 1,400 metabolites were quantified in plasma samples using the Ultrahigh Performance Liquid Chromatography-Tandem Mass Spectroscopy platform, which is a proprietary technology developed by Metabolon, Inc. (Durham, NC, USA) (Chen et al., 2023). For this study, we utilized a GWAS dataset specific to GBM that was sourced from the FinnGen Consortium R9 release data.^1^ This dataset originated from a comprehensive GWAS that was conducted on a cohort of individuals with European ancestry. This cohort consisted of 243 individuals diagnosed with GBM, while a control group of 287,137 participants (all cancers excluded) was utilized as a comparison. As the present study was based on publicly available summary data, no additional ethical approval or consent to participate was required.

### 2.3 Selection of IVs

Firstly, we excluded 15 bacterial taxa without specific names, leaving 196 bacterial traits, including 16 classes, 32 families, 119 genera, 20 orders and 9 phyla. We selected potential IVs for each exposure from the GWAS data based on SNPs that showed a genome-wide significant association (*p* < 5.0 × 10^−8^). Due to the limited number of available IVs, we adjusted the significance threshold to *p* < 1.0 × 10^−5^ for gut microbiota and metabolites. For the reverse MR analysis of GBM, we set the significance threshold to *p* < 5.0 × 10^−5^ to require obtain more than 20 SNPs. We then retained only the independent SNPs (window size>10 Mb, r^2^ < 0.001) based on the linkage disequilibrium structure of European populations. The harmonization process removed palindromic and incompatible alleles. To quantify the strength of IVs, we used the F-statistics, which were calculated using the following equation: F = R^2^(n-k-1)/k(1-R^2^), where R^2^ represented the proportion of variance in the exposure explained by the instrument, n was the sample size, and k represented the number of SNPs. A value of *F* > 10 indicated that there was no significant weak instrumental bias (Qu et al., 2024).

### 2.4 MR analysis

We used a bidirectional two-sample MR analysis to investigate the causal relationship between 196 bacterial traits and GBM. Several methods were used, including inverse variance weighted (IVW), MR Egger and weighted median. We used the IVW method as the main analysis because it provided the most reliable effect estimates and almost all the MR analyses used it as the main analysis (Chen et al., 2020; Larsson and Burgess, 2022; Xie et al., 2023). The other two methods were used as adjuncts or to observe whether their results were consistent with the direction of IVW. We selected the most significant bacterial taxa for further study. Sensitivity analysis and reverse MR were then performed to confirm the absence of horizontal pleiotropy and reverse causality. For reverse MR, the significance threshold for selecting IVs was set at *p* < 5.0 × 10^−5^. All other methods and settings were the same as those used in forward MR.

To select blood metabolites, we conducted a MR analysis to assess the causal relationship between the chosen bacterial taxon and 1,400 metabolites. We then included the significant metabolites as the exposure and used GBM as the outcome for the subsequent MR analysis. Finally, we selected the most significant metabolite as the mediator for the mediation MR analysis.

A mediation analysis was conducted using a two-step MR design to investigate whether plasma metabolites mediate the causal pathway from gut microbiota to GBM outcome (Figure 1B). The IVW approach was used to determine the total effect of gut microbiota on GBM (β), the effect of gut microbiota on metabolites (β1), and the effect of metabolites on GBM risk (β2). To calculate the indirect mediation effect of metabolites on GBM outcome, we used the coefficient difference method. This approach involves calculating the causal effect of gut microbiota on GBM via metabolites (β1 × β2) (Xu et al., 2022). We estimated the direct effect by adjusting for the mediator (β-β1 × β2). To calculate the percentage mediated, we divided the indirect effect by the total effect (β1 × β2/β) (Figure 1A).

### 2.5 Sensitivity analysis

The study utilized Cochran’s Q test from the IVW approach to evaluate the heterogeneity between the genetic variants (Yang et al., 2023). The MR-Egger regression intercept was used to detect the average horizontal pleiotropy (Qi et al., 2023). Lastly, the leave-one-out analysis and the MR-Pleiotropy Residual Sum and Outlier (MR-PRESSO) method were employed to assess whether outlier SNPs affected the causal association (Xu and Wang, 2023). The statistical analyses were conducted using the TwoSampleMR package (version 0.5.7) in R (version 4.2.1). A significance level of *p* < 0.05 was used to determine statistical significance.

## 3 Results

### 3.1 Genetic causality and correlation between gut microbiota and GBM

**Figure 2:**
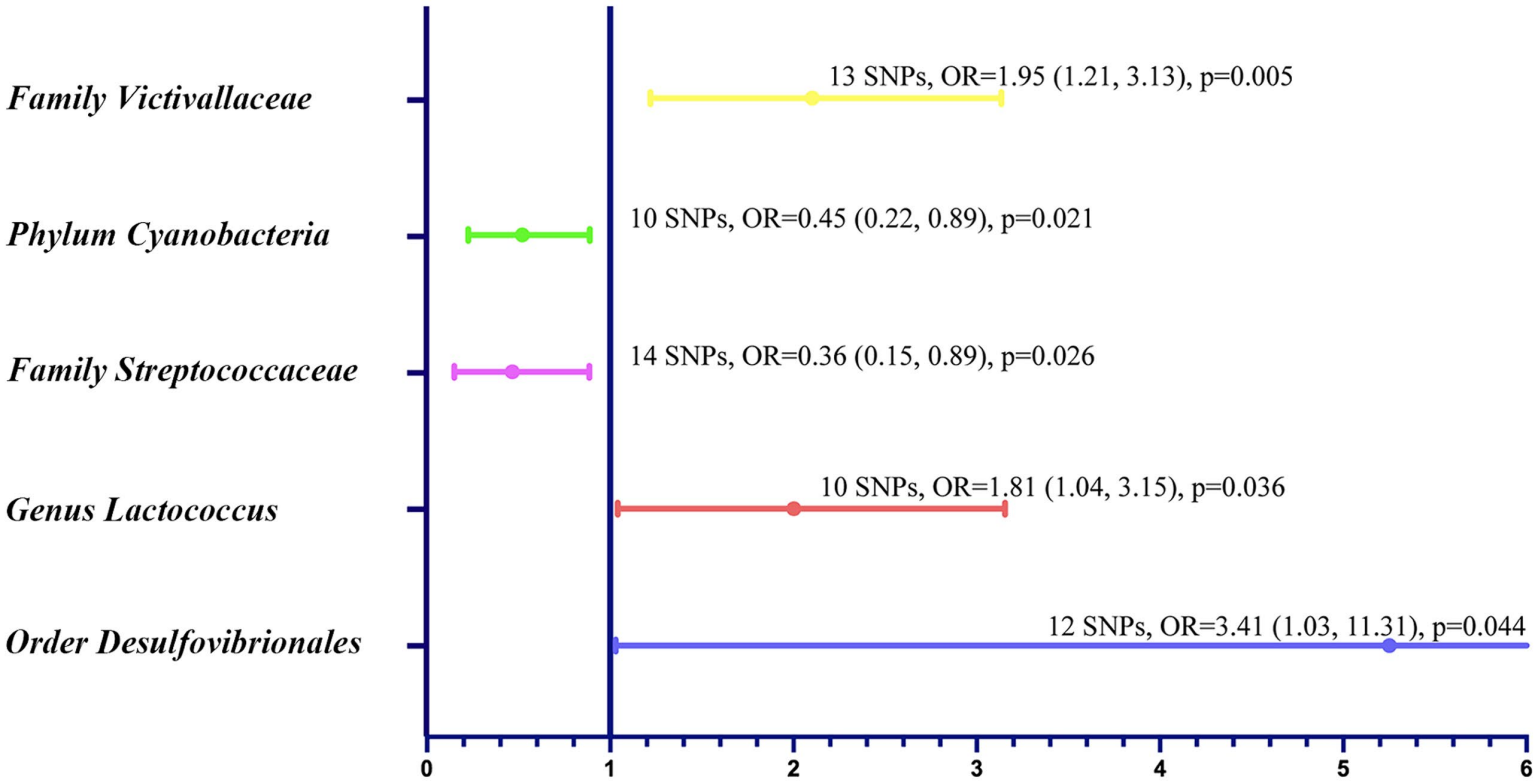
Forest plot to visualize the relationship between specific gut microbial taxa (exposure) and GBM (outcome).

In the bi-directional MR analysis of the causal relation between gut microbiota and GBM, we assessed 211 bacterial taxa. After eliminating 15 unknown taxa, we scrutinized the remaining 196, which yielded significant associations for five specific taxa (Figure 2). Notably, *families* such as *Streptococcaceae* and *Victivallaceae* demonstrated odds ratios (ORs) of 0.36 [95% confidence interval (CI): 0.15, 0.89; *p* = 0.026] and 1.95 (95% CI: 1.21, 3.13; *p* = 0.005), respectively. *Genus Lactococcus* showed an OR of 1.81 (95% CI: 1.04, 3.15; *p* = 0.036), and *order Desulfovibrionales* showed an OR of 3.41 (95% CI: 1.03, 11.31; *p* = 0.045). In the *phylum* category, *Cyanobacteria* had an OR of 0.45 (95% CI: 0.22, 0.89; *p* = 0.021). The Cochran’s Q test showed no significant heterogeneity of these IVs (Supplementary Table S1). The MR-PRESSO test did not detect any outliers (Supplementary Table S2), and the MR-Egger regression intercept analysis showed no potential directional horizontal pleiotropy (Supplementary Table S3). The scatter plots and leave-one-out plots for the causal association between these five microbial taxa and GBM were shown Supplementary Figure S1. No significant association was found between GBM and the five taxa in reverse MR analysis (Supplementary Table S4). For o*rder Desulfovibrionales*, the CIs around the IVW estimates were considerably wider and the *p* value was close to 0.05. Therefore, this taxon was not included in the further mediation analysis.

### 3.2 Mediator screening

In our study to identify the potential mediators, we initially explored the effect of gut microbiota on 1,400 plasma mediators. *Family Victivallaceae, genus Lactococcus, phylum Cyanobacteria* and *family Streptococcaceae* were found to be causally associated with 30, 50, 68 and 31 metabolites, respectively (Supplementary Tables S5–S8).

Following the exploration of the influence of gut microbiota on metabolites, we further examine the potential mediation effects of these significant mediators on GBM. For the *Victivallaceae-*associated metabolites, Methyl-4-hydroxybenzoate sulfate (M-4-HBS) (OR: 1.89; 95% CI: 1.14, 3.13; *p* = 0.013) and X-25790 (OR: 2.56; 95% CI: 1.30, 5.02; *p* = 0.006) had a positive effect on GBM. X-25790 was an unknown metabolite with little information, so the other metabolite M-4-HBS was included for mediation analysis.

*Lactococcus* showcased a notable mediation effect on GBM via two different mediators: phosphoethanolamine (PE) (OR: 1.62; 95% CI: 1.07, 2.45; *p* = 0.021) and phosphate to urate ratio (OR: 1.59; 95% CI: 1.03, 2.45; *p* = 0.036). PE had a smaller *p* value, so it was included in further analysis.

For the *Cyanobacteria*-associated metabolites, dehydroepiandrosterone sulfate (DHEAS) (OR: 1.55; 95% CI: 1.07, 2.25; *p* = 0.021) was the only metabolite that was correlated with GBM risk. As for *family Streptococcaceae*, no significant metabolite was found.

### 3.3 Mediation analysis of gut microbiota on GBM

**Figure 3:**
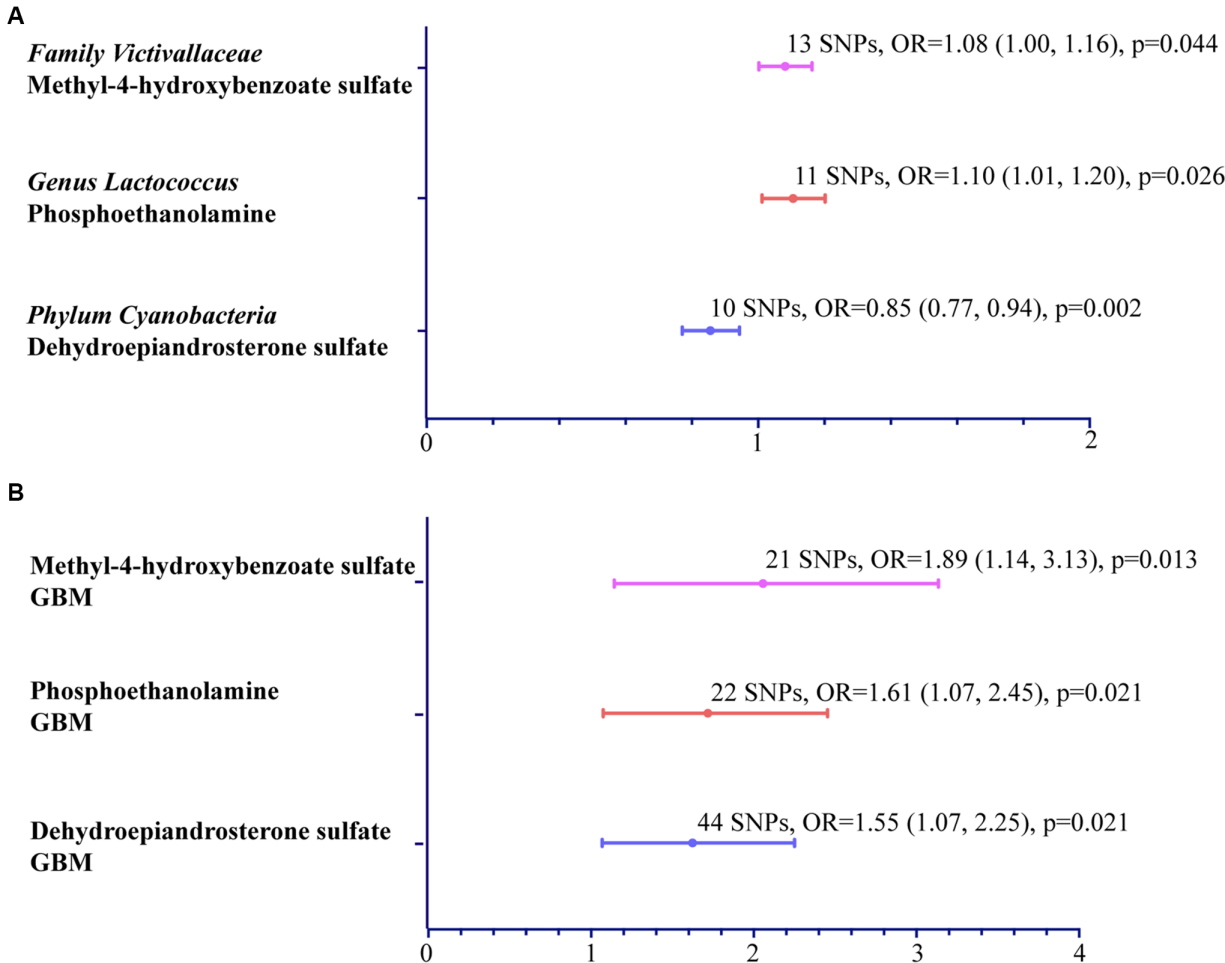
MR analysis: Microbiota’s effect on mediators and mediators’ on GBM. **(A)** Forest plot to visualize the effect between microbial taxa and specific mediators. **(B)** Forest plot exhibiting the causal effect of mediators on GBM.

As mentioned above, after mediator screening, we identified three pivotal metabolites: M-4-HBS, PE and DHEAS (Figure 3).

The study found a positive correlation between the presence of *family Victivallaceae* and M-4-HBS level (OR: 1.08; 95% CI: 1.00, 1.16; *p* = 0.044). Additionally, an elevated level of M-4-HBS was found to increase the likelihood of GBM (OR: 1.89; 95% CI: 1.14, 3.13; *p* = 0.013).

The *genus Lactococcus* was also found to have a positive association with PE (OR: 1.10; 95% CI: 1.01, 1.20; *p* = 0.026), which in turn was significantly associated with GBM (OR: 1.62, 95% CI: 1.07–2.45, *p* = 0.021).

The *phylum Cyanobacteria* showed a negative correlation with DHEAS level (OR: 0.85; 95% CI: 0.77, 0.94; *p* = 0.002). Conversely, increased DHEAS level was positively linked to GBM risk (OR: 1.55, 95% CI: 1.07, 2.25; *p* = 0.021).

### 3.4 Mediation proportion between gut microbiota and GBM

Mediation analysis was used to calculate the direct effect of specific gut microbiota on GBM (Table 1). *Family Victivallaceae* showed a mediation effect through M-4-HBS, which accounted for 7.27% of its total association with GBM. This was supported by a direct effect (β-β1 × β2) of 0.621 and a total effect (β) of 0.670. Similarly, *genus Lactococcus* demonstrated a significant mediation effect through PE level, accounting for approximately 7.98% of the total effect on GBM. The results indicate a direct effect (β-β1 × β2) of 0.547 and a total effect (β) of 0.594. Additionally, *phylum Cyanobacteria* showed a significant mediation effect through DHEAS level, accounting for 8.65% of its total influence on GBM. This was determined by a direct effect (β-β1 × β2) of −0.737 and a total effect (β) of −0.807.

**Table 1:** Mediation effect proportion.

Exposure	Mediator	Total effect (β)	Indirect effect (β1 × β2)	Direct effect (β-β1 × β2)	Proportion
*Family Victivallaceae*	M-4-HBS	0.670	0.049	0.621	7.27%
*Genus Lactococcus*	PE	0.594	0.047	0.547	7.98%
*Phylum Cyanobacteria*	DHEAS	−0.807	−0.070	−0.737	8.65%

M-4-HBS, Methyl-4-hydroxybenzoate sulfate; PE, phosphoethanolamine; DHEAS, dehydroepiandrosterone sulfate.

## 4 Discussion

The gut microbiota plays a critical role in the pathogenesis of GBM. Metabolites derived from the microbiota may function as key mediators of the disease (Lyu et al., 2022). Previous studies have demonstrated that glioma patients exhibit diminished microbial ecosystem diversity, with a notable overrepresentation of carcinogenic bacterial species including Fusobacterium and Akkermansia (Jiang et al., 2022). In addition, metabolites derived from gut microbiota, including SCFAs, glutamate, and tryptamines, may influence the immune environment and epigenetic landscape of GBM (Zelante et al., 2013; Fung et al., 2017; Baj et al., 2019). However, the causal relation between the GBM-related microbial taxa and metabolites has been rarely studied. Our study identified significant associations between specific microbial taxa and GBM, highlighting the potential role of gut microbiota in GBM progression. We also identified three blood metabolites associated with the three gut microbiota taxa and GBM using mediation analyses, suggesting a possible underlying mechanism. The study results indicate that *family Victivallaceae* and *genus Lactococcus* promote GBM by increasing the level of M-4-HBS and PE, respectively. In contrast, *phylum Cyanobacteria* exerts a protective effect against GBM via DHEAS. The exact relations and mediation effects could provide valuable insights into the potential therapeutic regimen targeting gut microbiota to manage GBM.

The study found that *family Victivallaceae* was a risk factor for GBM. This taxon has been linked to a higher risk of allergic rhinitis (Jin et al., 2023) and chronic obstructive pulmonary disease (Wei et al., 2023). However, the function of this bacterium is poorly understood, and there are no studies on its association with glioma (Jin et al., 2023). Therefore, this study provides a new perspective for bio-functional and mechanistic research on this bacterial taxon. The progression of GBM is associated with alterations in the gut-brain axis due to metabolites related to bacteria (Dehhaghi et al., 2020). One such metabolite is M-4-HBS, a common benzoate and xenobiotic metabolite, which has been found to significantly contribute to the prediction of brain failure (Bajaj et al., 2023). Studies have shown that this microbial-dependent metabolite was associated with brain dysfunction in patients with and without cirrhosis (Virdee et al., 2021), indicating a negative impact on CNS pathological conditions. Additionally, our study found that M-4-HBS was associated with high-grade glioma, and the presence of *Victivallaceae* was positively correlated with M-4-HBS, increasing the likelihood of GBM.

Our study demonstrated the positive role of *genus Lactococcus* in GBM initiation and progression. This taxon belongs to the lactic acid bacteria species and acts as a probiotic to exert beneficial effects on gut homeostasis (Gao et al., 2022). This bacterium shapes CNS function and host behavior via the gut-brain axis and has therefore been termed as “psychobiotics” (Dinan et al., 2013). *Lactococcus* has been reported to alleviate depressive and anxiety-like behaviors by restoring serotonin level in CNS (Gao et al., 2022; Ma et al., 2023b). Serotonin is a biogenic monoamine that acts as a neurotransmitter in CNS, a motility mediator in the gut and a vasoactive agent in the blood (Balakrishna et al., 2021). Although serotonin is primarily known for these functions, it has also been shown to activate GBM cell proliferation, migration, invasion and tumor angiogenesis (Sarrouilhe et al., 2021). In addition to serotonin, our mediation analysis identified PE as a vital mediator. PE is an intermediate in the synthesis of cell membrane phospholipids, with quantitative importance (Vance and Vance, 2004). Moreover, it has been demonstrated that PE itself or PE-derived ethanolamine is covalently linked to a diverse range of proteins, including those involved in signaling pathways, and to microtubule-associated protein 1 light chain 3 (LC3), which is essential for autophagosome formation (Vance and Tasseva, 2013). Recently, PE synthesized in mitochondria was found to be important for mitochondrial function (Johnson et al., 2023). The altered content of PE in membranes, as well as the levels of phospholipid metabolites and fatty acid profiles, are frequently identified as hallmarks of cancer development and progression (Stoica et al., 2022). GBM is characterized by active phospholipid metabolism (Viswanath et al., 2018), and elevated PE levels indicate increased membrane turnover to support cell proliferation. Prior clinic research demonstrated that isocitrate dehydrogenase 1 (IDH1) mutation altered phospholipid metabolism and resulted in decreased PE level in glioma. The level of PE may serve as an indicator for tumor-specific IDH1 status and a potential therapeutic target in the treatment of aberrant metabolic pathways in glioma (Esmaeili et al., 2014; Viswanath et al., 2018). The association between genus Lactococcus and GBM was mediated by PE. However, further randomized controlled trials are needed to explore the underlying mechanism.

*Cyanobacteria* was identified to be negatively associated with the risk of GBM. A previous case–control study revealed that bevacizumab-related treatments for patients with recurrent malignant gliomas were associated with reduced levels of *Cyanobacteria* in their fecal samples. This suggests that this particular microbial taxon was linked to therapeutic approaches in glioma patients (Zhu and Su, 2022). *In vitro* research demonstrated that these bacteria significantly suppressed the growth and migration of GBM cells, induced necrosis and lymphocytic infiltration, and decreased angiogenesis in GBM tissue (Arab et al., 2021). The antitumor effect of this bacterium can be attributed to the presence of beneficial metabolites. In addition, other bioactive components of *Cyanobacteria*, such as phycobiliprotein, phycocyanin and polysaccharide have gained considerable attention for their potential to prevent glioma carcinogenesis (Kawanishi et al., 2013; Syrpas et al., 2020; Arab et al., 2021). Our MR study provided genetic evidence that several specific serum metabolites mediated the causal effects of *Cyanobacteria* on GBM. In mediation analyses, the presence of *Cyanobacteria* was found to be negatively correlated with serum DHEAS level. Meanwhile, higher serum level of DHEAS was associated with a higher risk of GBM. A previous study found that the neurosteroid DHEAS could significantly reduce cell damage in neuroblastoma-glioma hybrid cells by attenuating oxidative stress (Lin et al., 2021). Additionally, DHEAS was found to be increased in temozolomide-resistant GBM cells and induced temozolomide resistance in GBM (Tetich et al., 2003). Although our data support some previous observations on the antineurotoxic action of DHEAS, further research is still needed to investigate the underlying biological mechanism.

Nevertheless, our study has several limitations. The exposure data only goes down to the genus level, which prevents us from investigating the causal relationship at a more specialized level, such as the species or strain level. If future microbiota GWASs use more advanced shotgun metagenomic sequencing analysis, the results may be more accurate and specific. Although the majority of participants in this GWSA were of European descent, it is important to note that population stratification may still have an impact on the results. Therefore, the findings of this study may not be applicable to individuals of non-European descent. The sample size of gut microbiota was relatively limited, which could potentially impact the results of the reverse MR analysis. It cannot be ruled out that GBM may affect the intestinal flora and the reverse causality needs to be confirmed by further studies with large simple size. Furthermore, our study is limited by the lack of multiple corrections due to the limited number of IVs available for the gut microbiota of interest and due to the small case number of GBM in the FinnGen R9 study. This limitation could be addressed in future research with a larger sample size for both the exposure and outcome variables. The IVs were selected at a significance threshold of *p* < 1 × 10^−5^, which is higher than the traditional GWAS threshold of *p* < 1 × 10^−8^, in order to obtain an adequate number of IVs. In future studies, we will expand the sample as much as possible to explore the association between gut microbiota and GBM. This will provide more theoretical support for the mechanism study of the gut-brain axis.

## 5 Conclusion

A total of five taxa demonstrated statistically significant associations with GBM. Among the identified taxa, f*amily Victivallaceae* and *genus Lactococcus* demonstrated a positive correlation with the risk of GBM. In contrast, the *phylum Cyanobacteria* exhibited a protective effect against GBM. Mediation analysis demonstrated that M-4-HBS, PE, and DHEAS served as intermediaries in the associations between *family Victivallaceae, genus Lactococcus, phylum Cyanobacteria*, and GBM. This study comprehensively assesses the association between gut microbiota, blood metabolites, and GBM. The identified bacterial strains may serve as novel biomarkers and provide clues for GBM pathogenesis. These findings offer new insights into microbiome-based therapies and metabolite-targeted interventions for GBM.

## Data availability statement

Publicly available datasets were analyzed in this study. This data can be found at: The datasets analyzed in our study are available in MiBioGen repository (https://mibiogen.gcc.rug.nl/), GWAS Catalog (https://www.ebi.ac.uk/gwas/), and FinnGen repository (https://r9.finngen.fi/).

## Ethics statement

Ethical approval was not required for the study involving humans in accordance with the local legislation and institutional requirements. Written informed consent to participate in this study was not required from the participants in accordance with the national legislation and the institutional requirements.

## Author contributions

XC: Data curation, Formal analysis, Writing – original draft. LH: Methodology, Software, Validation, Writing – review & editing. WX: Writing – review & editing, Funding acquisition, Project administration, Supervision.

## Footnotes

^1^ https://r9.finngen.fi/
