# Supplementary material for: Dissecting causal relationships between gut microbiota, blood metabolites, and glioblastoma multiforme: a two-sample Mendelian randomization study
Source: Front Microbiol. 2024 Jul 3;15:1403316. doi: 10.3389/fmicb.2024.1403316 (PMC11251919; doi:10.3389/fmicb.2024.1403316)
Supplement: Supplementary file 1 [file Table_1.DOCX]

Supplementary Material

# Supplementary Figures and Tables

## Supplementary Table S1

**The heterogeneity of gut microbiota instrumental variables**

| **Bacterial taxa (exposure)** | **Cochran's Q** | **Df** | ***P*-value** |
| --- | --- | --- | --- |
| *family Victivallaceae* | 13.21 | 12 | 0.354 |
| *phylum Cyanobacteria* | 8.44 | 9 | 0.491 |
| *family Streptococcaceae* | 10.54 | 13 | 0.649 |
| *genus Lactococcus* | 9.39 | 9 | 0.402 |
| *order Desulfovibrionales* | 16.65 | 11 | 0.119 |

Df, degree of freedom

## Supplementary Table S2

**MR-PRESSO analysis for the 5 bacterial traits that have been identified to be associated with the risk of GBM**

| **Bacterial taxa (exposure)** | **MR Analysis** | **Causal Estimate** | **SD** | **T** | ***P*-value** | **RSS_obs_** | **Global test *P*-value** | **Remove SNP** |
| --- | --- | --- | --- | --- | --- | --- | --- | --- |
| *family Victivallaceae* | MR-PRESSO | 0.67 | 0.24 | 2.78 | 0.017 | 15.77 | 0.373 | None |
| *phylum Cyanobacteria* | MR-PRESSO | -0.81 | 0.34 | -2.37 | 0.041 | 11.27 | 0.480 | None |
| *family Streptococcaceae* | MR-PRESSO | -1.01 | 0.41 | -2.47 | 0.028 | 12.40 | 0.646 | None |
| *genus Lactococcus* | MR-PRESSO | 0.594 | 0.28 | 2.10 | 0.065 | 11.72 | 0.426 | None |
| *order Desulfovibrionales* | MR-PRESSO | 1.23 | 0.61 | 2.01 | 0.070 | 19.79 | 0.145 | None |

﻿MR, Mendelian randomization; MR-PRESSO analysis, MR Pleiotropy RESidual Sum and Outlier analysis; GBM, glioblastoma multiforme; SD, standard deviation; RSS_obs_, observed residual sum of squares; SNP, single nucleotide polymorphism**﻿﻿**

## Supplementary Table S3

**Directional horizontal pleiotropy assessed by intercept term in MR Egger regression of the association between gut microbiota and GBM**

| **Bacterial taxa (exposure)** | **Egger intercept** | **SE** | ***P*-value** |
| --- | --- | --- | --- |
| *family Victivallaceae* | 0.24 | 0.16 | 0.176 |
| *phylum Cyanobacteria* | 0.02 | 0.14 | 0.879 |
| *family Streptococcaceae* | 0.07 | 0.14 | 0.656 |
| *genus Lactococcus* | 0.11 | 0.21 | 0.608 |
| *order Desulfovibrionales* | 0.23 | 0.17 | 0.199 |

MR, Mendelian randomization; GBM, glioblastoma multiforme; SE, standard error

## ﻿﻿ Supplementary Table S4

**Reverse MR estimates for the association between GBM and gut microbiota**

| **Bacterial taxa (outcome)** | **Methods** | **No. of SNPs** | **OR** | **95% CI** | ***P*-value** |
| --- | --- | --- | --- | --- | --- |
| *family Victivallaceae* | IVW | 23 | 1.02 | 0.99-1.04 | 0.172 |
| *phylum Cyanobacteria* | IVW | 26 | 1.00 | 0.98-1.02 | 0.996 |
| *family Streptococcaceae* | IVW | 27 | 1.00 | 0.99-1.01 | 0.942 |
| *genus Lactococcus* | IVW | 23 | 1.02 | 0.99-1.05 | 0.188 |
| *order Desulfovibrionales* | IVW | 27 | 1.01 | 1.00-1.03 | 0.158 |

MR, Mendelian randomization; GBM, glioblastoma multiforme; SNP, single nucleotide polymorphism; OR, odds ratio; CI, confidence interval; IVW, inverse variance weighted

## ﻿﻿ Supplementary Table S5

**Result of MR estimates for the association between *family Victivallaceae* and metabolites**

| **Bacterial taxa (exposure)** | **Metabolites (outcome)** | ***P*-value** |
| --- | --- | --- |
| *Family Victivallaceae* | 5,6-dihydrothymine levels | 0.039 |
| *Family Victivallaceae* | X-23593 levels | 0.026 |
| *Family Victivallaceae* | N-lactoyl tyrosine levels | 0.017 |
| *Family Victivallaceae* | Furaneol sulfate levels | 0.043 |
| *Family Victivallaceae* | X-25790 levels | 0.046 |
| *Family Victivallaceae* | Methyl-4-hydroxybenzoate sulfate levels | 0.044 |
| *Family Victivallaceae* | Benzoate to linoleoyl-arachidonoyl-glycerol (18:2 to 20:4) [1] ratio | 0.019 |
| *Family Victivallaceae* | 2-naphthol sulfate levels | 0.042 |
| *Family Victivallaceae* | Isoursodeoxycholate levels | 0.049 |
| *Family Victivallaceae* | N-methyltaurine levels | 0.014 |
| *Family Victivallaceae* | 5-hydroxyindole sulfate levels | 0.049 |
| *Family Victivallaceae* | X-21286 levels | 0.050 |
| *Family Victivallaceae* | N6-methyllysine levels | 0.008 |
| *Family Victivallaceae* | X-12112 levels | 0.009 |
| *Family Victivallaceae* | N2-acetyl,N6,N6-dimethyllysine levels | 0.025 |
| *Family Victivallaceae* | N2-acetyl,N6-methyllysine levels | 0.046 |
| *Family Victivallaceae* | Linoleoyl-arachidonoyl-glycerol (18:2/20:4) [1] levels | 0.034 |
| *Family Victivallaceae* | Alpha-ketoglutarate to glutamate ratio | 0.043 |
| *Family Victivallaceae* | Aspartate to citrulline ratio | 0.009 |
| *Family Victivallaceae* | 2-palmitoleoyl-GPC (16:1) levels | 0.009 |
| *Family Victivallaceae* | X-13729 levels | 0.005 |
| *Family Victivallaceae* | X-17654 levels | 0.018 |
| *Family Victivallaceae* | Alpha-ketoglutarate levels | 0.041 |
| *Family Victivallaceae* | X-12216 levels | 0.022 |
| *Family Victivallaceae* | Phosphate to oleoyl-linoleoyl-glycerol (18:1 to 18:2) [2] ratio | 0.044 |
| *Family Victivallaceae* | Retinol (Vitamin A) to linoleoyl-arachidonoyl-glycerol (18:2 to 20:4) [1] ratio | 0.045 |
| *Family Victivallaceae* | Retinol (Vitamin A) to oleoyl-linoleoyl-glycerol (18:1 to 18:2) [2] ratio | 0.009 |
| *Family Victivallaceae* | 2,6-dihydroxybenzoic acid levels | 0.025 |
| *Family Victivallaceae* | Adenosine 5'-diphosphate (ADP) to flavin adenine dinucleotide (FAD) ratio | 0.023 |
| *Family Victivallaceae* | Alpha-ketoglutarate to aspartate ratio | 0.016 |

MR, Mendelian randomization

## ﻿﻿ Supplementary Table S6

**Result of MR estimates for the association between *genus Lactococcus* and metabolites**

| **Bacterial taxa (exposure)** | **Metabolites (outcome)** | ***P*-value** |
| --- | --- | --- |
| *Genus Lactococcus* | Hexadecenedioate (C16:1-DC) levels | 0.039 |
| *Genus Lactococcus* | Octadecenedioate (C18:1-DC) levels | 0.014 |
| *Genus Lactococcus* | Cholesterol levels | 0.044 |
| *Genus Lactococcus* | Sphinganine levels | 0.037 |
| *Genus Lactococcus* | Gamma-glutamylglutamate levels | 0.046 |
| *Genus Lactococcus* | Taurocholic acid levels | 0.006 |
| *Genus Lactococcus* | Taurochenodeoxycholate levels | 0.008 |
| *Genus Lactococcus* | Glycochenodeoxycholate levels | 0.000 |
| *Genus Lactococcus* | Glycocholate levels | 0.001 |
| *Genus Lactococcus* | Pristanate levels | 0.045 |
| *Genus Lactococcus* | X-23659 levels | 0.025 |
| *Genus Lactococcus* | Phosphate to urate ratio | 0.036 |
| *Genus Lactococcus* | Eicosenoylcarnitine (C20:1) levels | 0.044 |
| *Genus Lactococcus* | Phosphoethanolamine levels | 0.026 |
| *Genus Lactococcus* | 2R,3R-dihydroxybutyrate levels | 0.044 |
| *Genus Lactococcus* | 1-(1-enyl-palmitoyl)-2-palmitoyl-GPC (P-16:0/16:0) levels | 0.028 |
| *Genus Lactococcus* | X-25828 levels | 0.039 |
| *Genus Lactococcus* | 3-(3-hydroxyphenyl) propionate sulfate levels | 0.019 |
| *Genus Lactococcus* | Octadecenedioylcarnitine (C18:1-DC) levels | 0.019 |
| *Genus Lactococcus* | 3-methoxycatechol sulfate (1) levels | 0.009 |
| *Genus Lactococcus* | Glycochenodeoxycholate 3-sulfate levels | 0.011 |
| *Genus Lactococcus* | Taurocholate to oxalate (ethanedioate) ratio | 0.020 |
| *Genus Lactococcus* | Glucuronate to androsterone glucuronide ratio | 0.026 |
| *Genus Lactococcus* | Salicylate to taurocholate ratio | 0.011 |
| *Genus Lactococcus* | Citrate to taurocholate ratio | 0.002 |
| *Genus Lactococcus* | 1,2-dipalmitoyl-gpc (16:0/16:0) levels | 0.005 |
| *Genus Lactococcus* | Cysteine s-sulfate levels | 0.009 |
| *Genus Lactococcus* | Theobromine levels | 0.019 |
| *Genus Lactococcus* | Glycerophosphoethanolamine levels | 0.034 |
| *Genus Lactococcus* | 1-palmitoyl-2-stearoyl-gpc (16:0/18:0) levels | 0.024 |
| *Genus Lactococcus* | 1-(1-enyl-stearoyl)-2-oleoyl-GPE (p-18:0/18:1) levels | 0.024 |
| *Genus Lactococcus* | X-17685 levels | 0.041 |
| *Genus Lactococcus* | Glycosyl-N-behenoyl-sphingadienine (d18:2/22:0) levels | 0.031 |
| *Genus Lactococcus* | Cholate to taurocholate ratio | 0.024 |
| *Genus Lactococcus* | Adenosine 3',5'-cyclic monophosphate (cAMP) to taurocholate ratio | 0.001 |
| *Genus Lactococcus* | Cysteine-glutathione disulfide levels | 0.020 |
| *Genus Lactococcus* | X-17328 levels | 0.019 |
| *Genus Lactococcus* | Gamma-glutamylglutamine levels | 0.043 |
| *Genus Lactococcus* | 1-palmitoyl-GPC (16:0) levels | 0.032 |
| *Genus Lactococcus* | N-acetylproline levels | 0.005 |
| *Genus Lactococcus* | X-11787 levels | 0.046 |
| *Genus Lactococcus* | Theophylline to theobromine ratio | 0.029 |
| *Genus Lactococcus* | Cholesterol to cortisol ratio | 0.021 |
| *Genus Lactococcus* | Cholesterol to taurocholate ratio | 0.029 |
| *Genus Lactococcus* | Cortisol to taurocholate ratio | 0.002 |
| *Genus Lactococcus* | Taurochenodeoxycholic acid 3-sulfate levels | 0.017 |
| *Genus Lactococcus* | Thyroxine to taurocholate ratio | 0.032 |
| *Genus Lactococcus* | Spermidine to taurocholate ratio | 0.004 |
| *Genus Lactococcus* | Choline to taurocholate ratio | 0.011 |
| *Genus Lactococcus* | Bilirubin (Z, Z) to taurocholate ratio | 0.006 |

MR, Mendelian randomization

## ﻿﻿ Supplementary Table S7

**Result of MR estimates for the association between *phylum Cyanobacteria* and metabolites**

| **Bacterial taxa (exposure)** | **Metabolites (outcome)** | ***P*-value** |
| --- | --- | --- |
| *Phylum Cyanobacteria* | Pregnenediol disulfate (C21H34O8S2) levels | 0.016 |
| *Phylum Cyanobacteria* | Androstenediol (3beta, 17beta) disulfate (1) levels | 0.048 |
| *Phylum Cyanobacteria* | Laurate (12:0) levels | 0.023 |
| *Phylum Cyanobacteria* | Arachidonate (20:4n6) levels | 0.031 |
| *Phylum Cyanobacteria* | Stearate (18:0) levels | 0.005 |
| *Phylum Cyanobacteria* | Pristanate levels | 0.043 |
| *Phylum Cyanobacteria* | Phytanate levels | 0.001 |
| *Phylum Cyanobacteria* | X-24546 levels | 0.012 |
| *Phylum Cyanobacteria* | X-24544 levels | 0.012 |
| *Phylum Cyanobacteria* | X-21470 levels | 0.029 |
| *Phylum Cyanobacteria* | Arachidoylcarnitine (C20) levels | 0.033 |
| *Phylum Cyanobacteria* | Docosatrienoate (22:3n6) levels | 0.045 |
| *Phylum Cyanobacteria* | Glucuronide of piperine metabolite C17H21NO3 (3) levels | 0.017 |
| *Phylum Cyanobacteria* | Caprate (10:0) levels | 0.016 |
| *Phylum Cyanobacteria* | N-succinyl-phenylalanine levels | 0.022 |
| *Phylum Cyanobacteria* | Phosphoethanolamine levels | 0.033 |
| *Phylum Cyanobacteria* | Androstenediol (3beta, 17beta) disulfate (2) levels | 0.004 |
| *Phylum Cyanobacteria* | Andro steroid monosulfate C19H28O6S (1) levels | 0.042 |
| *Phylum Cyanobacteria* | Pregnenediol sulfate (C21H34O5S) levels | 0.014 |
| *Phylum Cyanobacteria* | 16a-hydroxy DHEA 3-sulfate levels | 0.004 |
| *Phylum Cyanobacteria* | Androstenediol (3beta, 17beta) monosulfate (1) levels | 0.016 |
| *Phylum Cyanobacteria* | (16 or 17)-methylstearate (a19:0 or i19:0) levels | 0.005 |
| *Phylum Cyanobacteria* | 1-linoleoyl-2-arachidonoyl-GPC (18:2/20:4n6) levels | 0.003 |
| *Phylum Cyanobacteria* | Glycosyl-N-palmitoyl-sphingosine (d18:1/16:0) levels | 0.012 |
| *Phylum Cyanobacteria* | 1-linolenoyl-GPC (18:3) levels | 0.020 |
| *Phylum Cyanobacteria* | N-formylanthranilic acid levels | 0.016 |
| *Phylum Cyanobacteria* | 17alpha-hydroxypregnanolone glucuronide levels | 0.038 |
| *Phylum Cyanobacteria* | Octadecenedioylcarnitine (C18:1-DC) levels | 0.043 |
| *Phylum Cyanobacteria* | 9-hydroxystearate levels | 0.033 |
| *Phylum Cyanobacteria* | N-formylphenylalanine levels | 0.016 |
| *Phylum Cyanobacteria* | 5alpha-androstan-3alpha, 17beta-diol monosulfate (2) levels | 0.016 |
| *Phylum Cyanobacteria* | Propionylglycine levels | 0.027 |
| *Phylum Cyanobacteria* | Adrenate (22:4n6) levels | 0.028 |
| *Phylum Cyanobacteria* | Docosapentaenoate n3 DPA; 22:5n3 levels | 0.045 |
| *Phylum Cyanobacteria* | Docosahexaenoate DHA; 22:6n3 levels | 0.020 |
| *Phylum Cyanobacteria* | Stearidonate (18:4n3) levels | 0.027 |
| *Phylum Cyanobacteria* | DHEAS levels | 0.002 |
| *Phylum Cyanobacteria* | 3-hydroxymyristate levels | 0.040 |
| *Phylum Cyanobacteria* | Sulfate of piperine metabolite C16H19NO3 (3) levels | 0.028 |
| *Phylum Cyanobacteria* | Maltotriose levels | 0.025 |
| *Phylum Cyanobacteria* | N-alpha-acetylornithine levels | 0.035 |
| *Phylum Cyanobacteria* | Succinimide levels | 0.050 |
| *Phylum Cyanobacteria* | 3b-hydroxy-5-cholenoic acid levels | 0.031 |
| *Phylum Cyanobacteria* | 2-aminoheptanoate levels | 0.031 |
| *Phylum Cyanobacteria* | Arachidate (20:0) levels | 0.026 |
| *Phylum Cyanobacteria* | N-palmitoylglycine levels | 0.018 |
| *Phylum Cyanobacteria* | Dihomo-linolenate (20:3n3 or n6) levels | 0.031 |
| *Phylum Cyanobacteria* | 2-palmitoyl-GPC (16:0) levels | 0.048 |
| *Phylum Cyanobacteria* | 2-hydroxypalmitate levels | 0.012 |
| *Phylum Cyanobacteria* | 1-methylxanthine levels | 0.042 |
| *Phylum Cyanobacteria* | 1-arachidonylglycerol (20:4) levels | 0.024 |
| *Phylum Cyanobacteria* | X-16397 levels | 0.047 |
| *Phylum Cyanobacteria* | N-acetyl-aspartyl-glutamate (naag) levels | 0.031 |
| *Phylum Cyanobacteria* | Butyrate/isobutyrate (4:0) levels | 0.010 |
| *Phylum Cyanobacteria* | 2-hydroxystearate levels | 0.001 |
| *Phylum Cyanobacteria* | Dihomo-linoleate (20:2n6) levels | 0.040 |
| *Phylum Cyanobacteria* | Nonadecanoate (19:0) levels | 0.004 |
| *Phylum Cyanobacteria* | Margarate (17:0) levels | 0.019 |
| *Phylum Cyanobacteria* | Inosine to theophylline ratio | 0.039 |
| *Phylum Cyanobacteria* | Choline phosphate to choline ratio | 0.028 |
| *Phylum Cyanobacteria* | Caffeine to theophylline ratio | 0.045 |
| *Phylum Cyanobacteria* | Mannose to glycerol ratio | 0.025 |
| *Phylum Cyanobacteria* | Caffeine to theobromine ratio | 0.030 |
| *Phylum Cyanobacteria* | Carnitine to propionylcarnitine (C3) ratio | 0.038 |
| *Phylum Cyanobacteria* | 4-methylhexanoylglutamine levels | 0.038 |
| *Phylum Cyanobacteria* | Pregnenetriol disulfate levels | 0.015 |
| *Phylum Cyanobacteria* | Pregnenetriol sulfate levels | 0.002 |
| *Phylum Cyanobacteria* | Spermidine to (N (1) + N (8))-acetylspermidine ratio | 0.038 |
| *Phylum Cyanobacteria* | Pregnenediol disulfate (C21H34O8S2) levels | 0.016 |

MR, Mendelian randomization

## Supplementary Table S8

**Result of MR estimates for the association between *family Streptococcaceae* and metabolites**

| **Bacterial taxa (exposure)** | **Metabolites (outcome)** | ***P*-value** |
| --- | --- | --- |
| *Family Streptococcaceae* | Ceramide (d18:1/17:0, d17:1/18:0) levels | 0.006 |
| *Family Streptococcaceae* | Gluconate levels | 0.044 |
| *Family Streptococcaceae* | X-23659 levels | 0.050 |
| *Family Streptococcaceae* | X-24531 levels | 0.025 |
| *Family Streptococcaceae* | X-21607 levels | 0.048 |
| *Family Streptococcaceae* | Cerotoylcarnitine (C26) levels | 0.044 |
| *Family Streptococcaceae* | 3-indoleglyoxylic acid levels | 0.032 |
| *Family Streptococcaceae* | 2-hydroxyglutarate levels | 0.042 |
| *Family Streptococcaceae* | X-24736 levels | 0.021 |
| *Family Streptococcaceae* | X-25790 levels | 0.039 |
| *Family Streptococcaceae* | Ferulic acid 4-sulfate levels | 0.021 |
| *Family Streptococcaceae* | Glucose to N-palmitoyl-sphinganine (d18:0 to 16:0) ratio | 0.041 |
| *Family Streptococcaceae* | Bilirubin (Z,Z) to etiocholanolone glucuronide ratio | 0.033 |
| *Family Streptococcaceae* | 1-stearoyl-GPI (18:0) levels | 0.020 |
| *Family Streptococcaceae* | N-acetylglycine levels | 0.031 |
| *Family Streptococcaceae* | Maltotriose levels | 0.017 |
| *Family Streptococcaceae* | Phenylacetate levels | 0.016 |
| *Family Streptococcaceae* | Indolin-2-one levels | 0.014 |
| *Family Streptococcaceae* | N-palmitoyl-sphinganine (d18:0/16:0) levels | 0.042 |
| *Family Streptococcaceae* | X-21286 levels | 0.006 |
| *Family Streptococcaceae* | N-acetyl-1-methylhistidine levels | 0.047 |
| *Family Streptococcaceae* | X-12798 levels | 0.019 |
| *Family Streptococcaceae* | N-palmitoyl-sphingadienine (d18:2/16:0) levels | 0.031 |
| *Family Streptococcaceae* | Adenosine 5'-monophosphate (AMP) to inosine 5'-monophosphate (IMP) ratio | 0.034 |
| *Family Streptococcaceae* | Citrulline to dimethylarginine (SDMA + ADMA) ratio | 0.048 |
| *Family Streptococcaceae* | Phenylacetylglutamine levels | 0.010 |
| *Family Streptococcaceae* | X-26054 levels | 0.034 |
| *Family Streptococcaceae* | N1-methyladenosine levels | 0.021 |
| *Family Streptococcaceae* | X-11850 levels | 0.045 |
| *Family Streptococcaceae* | Isoleucine to phosphate ratio | 0.042 |
| *Family Streptococcaceae* | Carnitine to ergothioneine ratio | 0.050 |

MR, Mendelian randomization

## Supplementary Figure S1


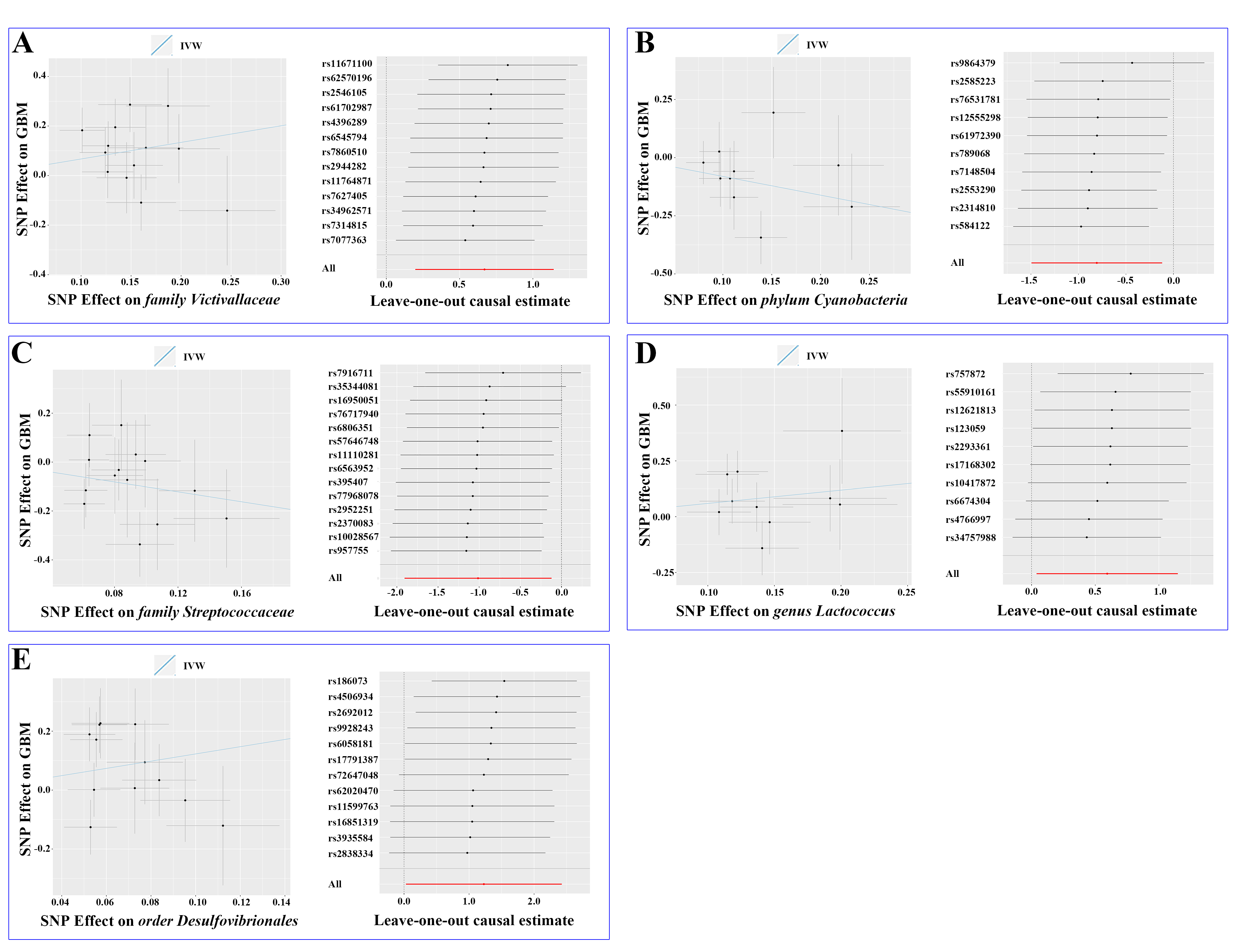


**Supplementary Figure S1.** Scatter plot and leave-one-out plot for the causal association between (A) *family Victivallaceae*, (B) *phylum Cyanobacteria*, (C) *family Streptococcaceae*, (D) *genus Lactococcus,* (E) *order Desulfovibrionales* and GBM.
